# Stem Cell Therapy in Neuroimmunological Diseases and Its Potential Neuroimmunological Complications

**DOI:** 10.3390/cells11142165

**Published:** 2022-07-11

**Authors:** Franz Felix Konen, Philipp Schwenkenbecher, Konstantin Fritz Jendretzky, Stefan Gingele, Lea Grote-Levi, Nora Möhn, Kurt-Wolfram Sühs, Britta Eiz-Vesper, Britta Maecker-Kolhoff, Corinna Trebst, Thomas Skripuletz, Martin W. Hümmert

**Affiliations:** 1 Department of Neurology, Hannover Medical School, Carl-Neuberg-Straße 1, 30625 Hannover, Germany; konen.felix@mh-hannover.de (F.F.K.); schwenkenbecher.philipp@mh-hannover.de (P.S.); jendretzky.konstantin@mh-hannover.de (K.F.J.); gingele.stefan@mh-hannover.de (S.G.); grote-levi.lea@mh-hannover.de (L.G.-L.); moehn.nora@mh-hannover.de (N.M.); suehs.kurt-wolfram@mh-hannover.de (K.-W.S.); trebst.corinna@mh-hannover.de (C.T.); skripuletz.thomas@mh-hannover.de (T.S.); 2 Institute of Transfusion Medicine and Transplant Engineering, Hannover Medical School, Carl-Neuberg-Straße 1, 30625 Hannover, Germany; eiz-vesper.britta@mh-hannover.de; 3 Department of Pediatric Hematology and Oncology, Hannover Medical School, Carl-Neuberg-Straße 1, 30625 Hannover, Germany; maecker.britta@mh-hannover.de

**Keywords:** stem-cell therapy, multiple sclerosis, NMOSD, MOGAD, myasthenia gravis, encephalitis, vasculitis, inflammatory polyneuropathy

## Abstract

**Background**: Since the 1990s, transplantations of hematopoietic and mesenchymal stem cells (HSCT and MSCT) and dendritic cell (DCT) have been investigated for the treatment of neurological autoimmune disorders (NADs). With the growing number of transplanted patients, awareness of neuroimmunolgical complications has increased. Therefore, an overview of SCT for the most common NADs and reports of secondary immunity after SCT is provided. **Methods**: For this narrative review, a literature search of the PubMed database was performed. A total of 86 articles reporting on different SCTs in NADs and 61 articles dealing with immune-mediated neurological complications after SCT were included. For multiple sclerosis (MS), only registered trials and phase I/II or II studies were considered, whereas all available articles on other disorders were included. The different transplantation procedures and efficacy and safety data are presented. **Results**: In MS patients, beneficial effects of HSCT, MSCT, and DCT with a decrease in disability and stabilization of disease activity have been reported. These effects were also shown in other NADs mainly in case reports. In seven of 132 reported patients with immune-mediated neurological complications, the outcome was fatal. **Conclusions**: Phase III trials are ongoing for MS, but the role of SCT in other NADs is currently limited to refractory patients due to occasional serious complications.

## 1. Introduction

Hematopoietic stem-cell transplantation (HSCT) is a complex treatment procedure, which was originally developed for treating hematological malignancies [1,2,3]. Although the exact mechanism of action has not yet been precisely defined, the rationale is to erase the aberrant cells with a conditioning regimen [2]. As an ancillary effect, the applied myelotoxic agents deplete bone marrow-inherent stem cells and reduce the mature lymphocyte pool [1,2]. Thereafter, patients’ own (autologous) or donors’ (allogeneic) hematopoietic stem cells harvested from peripheral blood or bone marrow are used to repopulate the depleted bone marrow and reconstitute the immune system without (autologous) or with (allogeneic) a graft-versus-autoreactivity effect [1,2,3].

In the 1990s, the reconstitution of a new immune system after hematopoietic stem-cell transplantation was first considered as treatment option for autoimmune-mediated disorders through the establishment of self-tolerance. Therefore, pilot studies investigated autologous HSCT in patients suffering from multiple sclerosis (MS), systemic sclerosis, rheumatoid arthritis, and systemic lupus erythematosus (SLE) [4,5,6,7]. Thereafter, several trials investigated the transplantation of stem cells of different origin in various autoimmune diseases. These included transplantation of mesenchymal stem cells, which may also be of autologous or allogeneic origin and derive from peripheral blood, bone marrow, adipose tissue (by lipectomy), or umbilical cord blood. Mesenchymal stem cells are intravenously or intrathecally applied and exert their effects via immunomodulatory and trophic mechanisms by secreting cytokines, growth factors, hormones, and microRNA, which are released as soluble molecules or as loaded extracellular vesicles [8]. Lately, intravenous application of autologous, peripheral blood-derived, peptide-loaded dendritic cells was proposed to induce self-tolerance in patients with autoimmune-mediated diseases [9].

Reported effects of stem-cell transplantation were the quantitative and qualitative restoration of T and B cells, as well as a change in cytokine and chemokine profiles, leading to a status favoring less autoimmunity, which was also observed in the central nervous system (CNS) [10,11,12,13]. Furthermore, an improvement of the safety and efficacy of stem-cell transplantations was achieved due to the increased experience of transplanting centers including improved transplant techniques and better selection of eligible patients [14,15]. Subsequently, an increase in stem-cell transplantations in patients with neurological autoimmune conditions was reported [15,16]. The European Society for Blood and Marrow Transplantation (EBMT) and the North American Center for International Blood and Marrow Transplant Research (CIBMTR) revealed that 40% of the hematopoietic stem-cell transplantations, which were indicated due to autoimmune disorders, were performed in patients with neurological diseases, mainly MS [15,16].

In general, the aim of any treatment of patients with autoimmune diseases is to achieve a long-term disease-free state. Although definitive long-term data are not available, study results with a longer observation period on the use of autologous HSCT in MS indicated that the probability of sustained remission was higher compared to conventional immunotherapies available at that time [17,18,19]. Taking into account possible long-term side effects of frequently administered disease-modifying therapies, particularly an increased risk of infection and malignancy, the achievement of a sustained therapeutic effect by a single HSCT treatment seems attractive [20]. Long-term studies need to clarify the benefit–risk ratio of both therapy modalities in a direct comparison, especially in view of the numerous highly potent new immunotherapeutics in the neuroimmunological field.

Despite the improved general safety of transplantation procedures, the rising numbers of stem-cell transplantations to treat hemato-oncological diseases, as well as neurological autoimmune disorders, increased the awareness of adverse events affecting the nervous system [21]. Interestingly, new onset of immune-mediated phenomena after stem-cell transplantation partly involving the nervous system was reported [22]. Although these secondary neuroimmunological complications are rare, they are associated with high morbidity and mortality, particularly if diagnosis is missed or delayed [23].

Therefore, the present review article provides an overview of the available stem-cell therapies for neurological autoimmune diseases in the era of emerging new immunotherapies and summarizes the available reports of immune-mediated neurological complications after stem-cell transplantation, of relevance to any stem-cell therapist and/or neurologist. Accordingly, we provide a comprehensive overview of the current status of the use of stem cells in the neuroimmunological diseases (a) MS, (b) NMOSD and MOGAD, (c) autoimmune-mediated encephalitis and vasculitis with CNS involvement, and (d) autoimmune-mediated diseases of the peripheral nervous system (chronic inflammatory demyelinating polyneuropathy, CIDP; multifocal motoric polyneuropathy, MMN; Guillain–Barré syndrome, GBS; myasthenia gravis). Thereafter, we focus on the topic of secondary immune-mediated neurological complications after stem-cell transplantation, which is highly relevant for clinicians and those affected.

## 2. Materials and Methods

The NIH National Library of Medicine PubMed.gov database (https://pubmed.ncbi.nlm.nih.gov/, latest access on 16 May 2022) was used for an extensive literature research concerning stem-cell therapies in autoimmune-mediated neurological diseases. Search terms were “stem-cell transplantation” and “stem-cell therapy” combined with “multiple sclerosis”, “neuromyelitis optica spectrum disorders”, “MOG antibody-associated disease” (MOGAD), “encephalitis”, “vasculitis”, “CIDP”, “MMN”, “neuropathy”, and “myasthenia gravis”. Applying these search terms, more than 2000 articles were found, which were screened for eligible articles. Due to the large number of eligible articles which were obtained for the search term “multiple sclerosis”, only registered clinical trials, phase I, I/II, and II studies, and respective post hoc analyses were considered for the present review article. For the other search terms, a lower number of eligible articles were available; thus, all of these were included. Furthermore, all articles, which were initially considered as relevant, were manually screened for eligible references. For MOGAD, only one case report published as an abstract could be considered as eligible for this review article.

## 3. Results and Discussion

In this section, studies and publications from more than 20 years of stem cell transplantation for the most common neurological autoimmune disorders are presented. During this time, new conditioning regimens, infused cells, and applications emerged. To summarize the data, partly wide ranges are given. Additionally, more detailed information is given in Supplementary Tables S1 and S2.

### 3.1. Stem-Cell Therapies in MS

A total of 44 phase I, I/II, and II studies, as well as registered clinical trials and post hoc analyses on cell therapy for MS, were found, and a summary of the results is shown in Table 1 (for further details, see Supplementary Table S1) [9,17,24,25,26,27,28,29,30,31,32,33,34,35,36,37,38,39,40,41,42,43,44,45,46,47,48,49,50,51,52,53,54,55,56,57,58,59,60,61,62,63,64,65,66,67,68]. The number of included patients in these studies varied between five and 617, with most investigating chronic–progressive forms of MS (five trials included relapsing–remitting MS patients only). The age of the included patients varied between 28 and 49 years with a disease duration of 2.6 to 20 years. Kurtzke’s expanded disability status scale (EDSS) score ranged between 3 and 7.5 (median 6) at treatment onset [69]. The study population was followed up between 1 and 80 months (excluding post hoc analyses).

In 24 of 43 studies, the results of autologous HSCT were reported, with a total of 1849 included patients [17,24,25,26,27,28,29,30,31,32,33,34,35,36,37,38,39,40,41,42,43,44,45,46]. In all of these studies, the hematopoietic stem cells were derived from peripheral blood [17,24,25,26,27,28,29,30,31,32,33,34,35,36,37,38,39,40,41,42,43,44,45,46]. Peripheral blood stem cells were mobilized using cyclophosphamide in combination with G-CSF (granulocyte-colony stimulating factor, *n* = 17), additional application of GM-CSF (granulocyte-macrophage colony-stimulating factor, *n* = 2), and G-CSF only (*n* = 4) [17,24,25,26,27,28,29,30,31,32,33,34,35,36,37,38,39,40,41,42,43,44,45,46]. In one study, hematopoietic stem cells originated from bone marrow (harvested by bone marrow aspiration), but insufficient transplantable cells were obtained; thus, peripheral blood stem cells (mobilized by cyclophosphamide and G-CSF) were also transplanted [42].

After HSCT (irrespective of the applied conditioning regimen), beneficial effects clinically defined as improvement (>1 point in EDSS) or stability (±0.5 points in EDSS) were reported in 16–100% of the included MS patients (Table 1) [17,24,25,26,27,28,29,30,31,32,33,34,35,36,37,38,39,40,41,42,43,44,45,46]. In some of these patients (*n* = 553) with beneficial effects, further outcome differentiation was possible; in 3–81%, EDSS improved (decreased) by at least one point, while it was stable (±0.5 compared with EDSS at treatment onset) in 12–100% [17,24,25,26,27,28,29,30,31,32,33,34,35,36,37,38,39,40,41,42,43,44,45,46]. Adverse events related to the treatment procedure were reported in all trials in which hematopoietic stem cells were infused [17,24,25,26,27,28,29,30,31,32,33,34,35,36,37,38,39,40,41,42,43,44,45,46]. Neutropenia-associated infections, toxicity associated with the conditioning regimen, and transient worsening of neurological symptoms were frequently stated. Treatment-related deaths occurred in seven studies (total of 11/584, 2%) [17,24,25,26,27,28,29,30,31,32,33,34,35,36,37,38,39,40,41,42,43,44,45,46].

In patients with disease progression and relapses despite stem-cell therapy, MRI revealed progressive brain atrophy and new lesions in T2-weighted and/or gadolinium-enhanced T1-weighted images. However, post hoc analyses revealed that progressive brain atrophy, which can be commonly found in untreated MS disease course, was slowed down after autologous hematopoietic stem-cell therapy [47,48] Contrarily, one post hoc analysis reported of discordant findings concerning disease activity [49]. Despite stable disease activity regarding MRI and cerebrospinal fluid parameters, clinical parameters (EDSS and ambulation) indicated worsening of the patients’ symptoms [49].

Furthermore, 19 studies reported the results following the transplantation of mesenchymal stem or stroma cells [50,51,52,53,54,55,56,57,58,59,60,61,62,63,64,65,66,67,68]. In 13 of these studies, autologous mesenchymal stem cells gained from bone marrow aspiration were used [50,51,52,53,54,55,56,57,58,59,60,61,62]. One study used autologous peripheral blood mesenchymal stem cells (mobilized by G-CSF), while two studies reported the usage of adipose-derived mesenchymal stem cells (derived from lipectomy) [63,64,65]. In contrast, three studies used allogeneic mesenchymal stem cells for transplantation [66,67,68]. These originated from either human umbilical cord blood, human placenta tissue, or Wharton’s jelly (gelatinous substance within the umbilical cord) [66,67,68]. As opposed to hematopoietic stem-cell transplantation, mesenchymal stem cells (MSCs) and stroma cells for the treatment of MS were applied intrathecally (*n* = 9), intravenously (*n* = 8), or using a combination of both applications (intravenously and intrathecally, *n* = 2) [50,51,52,53,54,55,56,57,58,59,60,61,62,63,64,65,66,67,68]. Since no conditioning regimen was applied for mesenchymal stem-cell transplantation, treatment-related adverse events were rarely recorded and were generally mild. Reported adverse events consisted in most cases of back pain and headache (intrathecal application), infections, and transient worsening of neurological symptoms [50,51,52,53,54,55,56,57,58,59,60,61,62,63,64,65,66,67,68]. Treatment-related deaths were not reported in any mesenchymal stem-cell therapy study (follow-up period 5.6–88.8 months) [50,51,52,53,54,55,56,57,58,59,60,61,62,63,64,65,66,67,68].

Beneficial treatment effects, clinically raised by EDSS improvement or stability, were reported in 18–100% of the included patients, while, in 0–50%, beneficial effects were not reported [50,51,52,53,54,55,56,57,58,59,60,61,62,63,64,65,66,67,68]. In some studies, differentiation of beneficial results between improvement and stabilization was possible. EDSS improvement (>1 point) was reported in 0–48% and EDSS stability (±0.5 points) was reported in 29–100% of the included patients [50,51,52,53,54,55,56,57,58,59,60,61,62,63,64,65,66,67,68].

Lastly, in one phase I study including eight patients suffering from relapsing–remitting (1 patient), primary progressive (three patients), and secondary progressive MS (four patients), peptide-loaded tolerogenic dendritic cells from peripheral blood were administered intravenously [9]. These cells were generated by conditioning monocyte-derived dendritic cells after autologous monocyte-derived dendritic cells were obtained by leukapheresis [9]. A conditioning regimen preceding intravenous application of these cells was not applied [9]. All included patients revealed stable EDSS scores during the short follow-up period of 3 months, and no treatment-related adverse events were reported [9].

**Figure 1:**
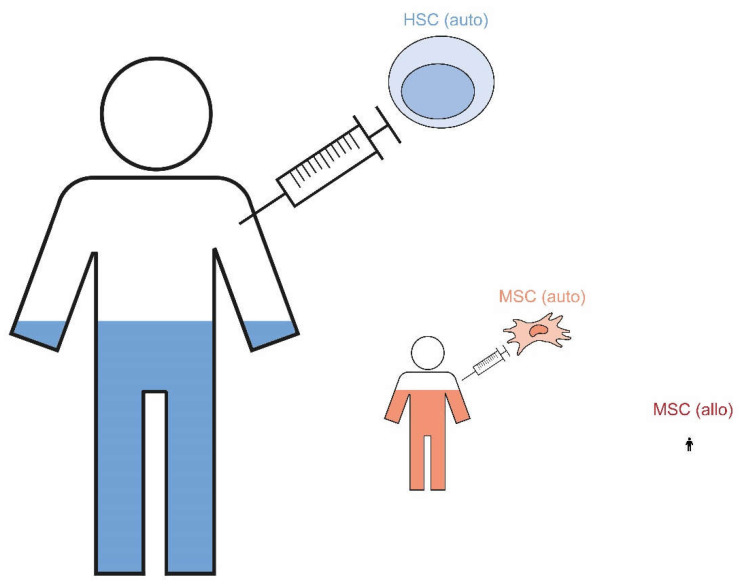
Due to the small number of patients who received peptide-loaded tolerogenic dendritic cells, they are not shown in the figure. HSC = hematopoietic stem cell; MSC = mesenchymal stem cell; auto = autologous; allo = allogeneic.

In summary, stem-cell therapies seem to effectively reduce disease activity and even restore neurological function in patients with MS. In particular, autologous HSCT with an intermediate intensity-conditioning regime is proposed as highly effective in reducing disease activity in MS patients. Burt and colleagues reported no evidence in disease activity (NEDA) in 93% of cases (within a median follow-up of 2 years; conditioning regime: cyclophosphamide + anti-thymocyte globulin (ATG)), whereas Nash and colleagues described NEDA in 69% of cases (median follow-up of 5 years; BEAM (carmustine, etoposide, cytarabine, and melphalan) + ATG) [70,71]. Similarly, this review indicates a wide range of beneficially affected MS patients after stem-cell therapies (Figure 1). Nevertheless, the results of the previously mentioned studies seem to indicate superiority of HSCT in reaching NEDA compared with other highly effective MS disease-modifying therapies (DMTs) such as ocrelizumab, alemtuzumab, or cladribine [72,73,74,75]. However, direct comparison of these data is not suitable since the trials differed in eligibility criteria, design, and follow-up duration, including prior DMT treatment and disease activity at study entry. In order to obtain clarity on this issue, both therapeutic approaches (HSCT vs. highly effective DMTs such as cladribine, alemtuzumab, and ocrelizumab) are currently being compared in various studies, some of which are phase III trials [76].

### 3.2. Stem-Cell Therapies in NMOSD and MOGAD

A total of 16 articles on stem-cell therapy in NMOSD, including case reports (*n* = 9), retrospective observational studies (*n* = 1), and phase I (*n* = 1), I/II (*n* = 4), and II (*n* = 1) studies, were found (see Table 2; for more details, see Supplementary Table S2) [77,78,79,80,81,82,83,84,85,86,87,88,89,90,91]. In the studies considered, a total of 73 patients (61 female, age 2–64 years, disease duration 1–11 years) suffering from NMOSD were included [77,78,79,80,81,82,83,84,85,86,87,88,89,90,91]. EDSS at treatment onset varied between 3.5 and 8.5 (median 5), and patients were followed up between 3 and 108 months [77,78,79,80,81,82,83,84,85,86,87,88,89,90,91]. In eight of these reports (two phase I/II studies), peripheral blood autologous hematopoietic stem cells were intravenously infused [77,78,79,80,81,82,83,84]. Stem cells were mobilized using cyclophosphamide and G-CSF with additional rituximab application in one study. In another study, bone marrow-derived hematopoietic stem cells were used [80]. 

Most investigations reported on beneficial treatment effects, with improvement or stability of clinical parameters assessed by EDSS and disease activity assessed by MRI [77,78,79,80,81,82,83,84,85]. Thus, Burt and colleagues reported EDSS improvement from a baseline of 4.4 to 3.3 after 5 years [78]. Contrarily, two case reports presented the absence of beneficial treatment effects with persistent relapsing activity and uncontrolled disease activity (MRI) [83,85]. In these patients, BEAM and cyclophosphamide + rituximab + ATG were chosen as conditioning regimens [83,85]. Almost all investigations reported treatment procedure-associated adverse events, most frequently infections. Furthermore, in the phase I/II study of Burt and colleagues, transient worsening of neurological symptoms due to treatment application was reported [78]. Among all NMOSD patients with HSCT, one treatment-related death was reported [79].

In three case reports with four patients, allogeneic hematopoietic stem cells were infused [86,87,88]. Similarly to the transplantation of autologous hematopoietic stem cells, mostly beneficial treatment effects were reported [86,87,88]. EDSS improvement of at least one point was reached in three out of four patients, and EDSS stability was reached in one out of four patients [86,87,88].

In addition, two phase I/II studies used umbilical cord blood-derived allogeneic mesenchymal stem cells as treatment for NMOSD [89,90]. The stem cells were applied intravenously and intrathecally in five patients each [89,90]. No conditioning regime was employed, and no severe treatment-related adverse events were reported [89,90]. Beneficial treatment effects (EDSS improvement or stability) were reported in three and four out of five of the included patients, respectively [89,90]. In two of these patients, relapses during follow-up (24 months and 70 months (mean)) were fatal and led to NMOSD-related death [89,90]. 

In one phase II study, bone marrow-derived autologous mesenchymal stem cells were intravenously applied in 15 patients suffering from NMOSD [91]. During a follow-up of 24 months, all patients reported of beneficial treatment effects displayed by an EDSS improvement >1 point (six patients) or EDSS stability ±0.5 points (nine patients) [91]. No adverse events or deaths related to the treatment procedure were reported [91].

Lastly, in one phase I study, four NMOSD patients received intravenously applied peptide-loaded tolerogenic dendritic cells [9]. Of these patients, one improved notably (EDSS improvement > 1) and three remained stable (EDSS ± 0.5) during the 3 month follow-up [9]. No treatment-related adverse events were reported during this short follow-up period [9].

There are no reports on stem-cell therapies in patients with MOGAD in the literature so far. One nonoriginal article reported a male patient with MOGAD who suffered from relapsing disease activity despite autologous HSCT [92].

In summary, stem-cell therapies in NMOSD and MOGAD patients play a rather experimental role [71]. To date, a total of 73 published patients have been treated with stem cell therapies, and there are no trials which directly compare classic immunotherapeutic treatment approaches and stem-cell therapies. Furthermore, there are different highly effective approved DMTs for NMOSD, including eculizumab, satralizumab, and inebilizumab [93,94,95]. In addition, other off-label therapeutics such as rituximab and tocilizumab have been used with success [96,97]. With respect to eculizumab, 94% patients were relapse-free in the extension study of the pivotal trial, even after more than 3.5 years [97]. However, this therapy in particular is extremely costly; hence, from a socioeconomic point of view and taking into account the burden on quality of life due to strictly required regular infusions, a possibly one-time stem-cell transplantation offers advantages [98]. Therefore, NMOSD and MOGAD patients should be studied only in randomized, actively controlled trials in the case of stem-cell therapy to evaluate the true value of this therapeutic approach in the era of highly effective immunotherapeutics.

### 3.3. Stem-Cell Therapies in Autoimmune-Mediated Encephalitis and Vasculitis with Affection of the CNS

Five case reports or case series including six patients were found which reported stem-cell transplantations in patients with autoimmune-mediated encephalitis and vasculitis with affection of the CNS:

One patient suffering from autoimmune-mediated encephalitis was a 35 year old woman with concomitant common variable immunodeficiency (CVID), who revealed uncontrolled disease activity with new MRI lesions despite application of different immunomodulatory therapies comprising intravenous immunoglobulins (IVIG), prednisolone, azathioprine, rituximab, cyclophosphamide, and abatacept [99]. Due to the highly refractory disease course, a double cerebral biopsy was performed, and the histologic results were compatible with autoimmune-mediated encephalitis (MS and lymphoma were excluded) [99]. 

Four female patients with systemic lupus erythematosus (SLE, age 18–25 years, disease duration 2–9 years) also suffered from CNS affection [100,101,102]. Neurological manifestations included paresis/plegia, loss of vision, disturbance of speech, bladder and bowel dysfunction, headache, and hallucinations [99,100,101,102]. Results of neurological diagnostic work-up revealed longitudinal extensive transverse myelitis (LETM), optic neuritis, cerebral lesions, and infarction [100,101,102]. Two of these patients may actually have had NMOSD according to the current diagnostic criteria [101,102]. Autologous hematopoietic stem cells were mobilized with cyclophosphamide and G-CSF in all of these patients except one SLE patient who underwent bone marrow aspiration [100,101,102]. Conditioning regimens were cyclophosphamide + ATG (*n* = 3), cyclophosphamide + total body irradiation (*n* = 1), and etoposite + melphalan (*n* = 1) [99,100,101,102]. Adverse events were reported in all patients, including infections, treatment-related toxicity, and transient worsening of neurological symptoms [99,100,101,102]. However, no deaths related to the treatment procedure were reported [99,100,101,102]. During follow-up (6–45 months), the disease course of all patients was beneficially affected (partly with complete recovery) [99,100,101,102]. MRI lesions of the patient suffering from autoimmune-mediated encephalitis completely resolved after autologous HSCT [99]. 

Lastly, Gray and colleagues reported a 9 year old boy with cerebral vasculitis in X-linked lymphoproliferative disease, who presented with polyfocal neurological deficits associated with multiple infarctions [103]. In this patient, allogeneic stem cells (umbilical cord blood derived) were transplanted after a reduced-dosage conditioning regimen with busulfan and fludarabine [103]. Adverse events included infections, treatment-related toxicity, transient worsening of neurological symptoms, and onset of secondary autoimmunity (anti-glomerular basement membrane (GBM) disease) [103]. Under treatment with corticosteroids, cyclophosphamide, and rituximab, anti-GBM disease was controlled with ongoing moderate renal function impairment [103]. During follow-up (13.8 months), clinical remission of cerebral vasculitis was reached, and MRI displayed stabilization of the disease [103].

In summary, reports of HSCT in patients suffering from autoimmune-mediated encephalitis or vasculitis with affection of the CNS are relatively scarce. Therefore, HSCT is rather to consider as an experimental approach. Nevertheless, the reported patients were refractory against treatment with a broad spectrum of anti-inflammatory therapies and beneficially responded to HSCT. In treatment-refractory patients with great disease burden, HSCT may be considered as ultima ratio therapy, especially in patients with underlying CVID.

### 3.4. Stem-Cell Therapies in Autoimmune-Mediated PNS Diseases

A total of 20 case reports and one phase I/II study investigating stem-cell therapies in 108 patients (37 women, age 17–75 years, disease duration 1–38 years, follow-up 7–78 months) suffering from CIDP, MMN, and myasthenia gravis were found [104,105,106,107,108,109,110,111,112,113,114,115,116,117,118,119,120,121,122,123,124]. Of these patients, 95 suffered from CIDP, 10 suffered from myasthenia gravis, and one each suffered from MMN, myasthenia gravis and concomitant amyotrophic lateral sclerosis (ALS), and myasthenia gravis and concomitant polymyositis [104,105,106,107,108,109,110,111,112,113,114,115,116,117,118,119,120,121,122,123,124]. Due to consideration of several case reports, the patients’ symptoms and disease-related disability were highly heterogeneous and scored with different clinical evaluation tools (modified Rankin scale, inflammatory neuropathy cause and treatment score, Rasch-built overall disability scale, medical research council sum score, myasthenia gravis foundation of America score, and myasthenia gravis composite score) [104,105,106,107,108,109,110,111,112,113,114,115,116,117,118,119,120,121,122,123,124]. On the one hand, there were patients, who were treated with stem-cell therapies due to the dependence of regular treatment (IVIG and plasmapheresis), but who responded well to this initial treatment and recovered completely [104,105,106,107,108,109,110,111,112,113,114,115,116,117,118,119,120,121,122,123,124]. These patients were not severely disabled, and symptoms mostly did not influence daily activities [104,105,106,107,108,109,110,111,112,113,114,115,116,117,118,119,120,121,122,123,124]. On the other hand, there were patients, who were treatment-refractory and/or highly disabled (including the need for intensive care and ventilation) [104,105,106,107,108,109,110,111,112,113,114,115,116,117,118,119,120,121,122,123,124]. In these patients, stem-cell therapy was offered as an ultima ratio treatment option [104,105,106,107,108,109,110,111,112,113,114,115,116,117,118,119,120,121,122,123,124]. However, of the 60 CIDP patients enrolled in the phase I/II study by Burt and colleagues, various clinical scoring tools indicated moderate disability and need for assistance with activities of daily living [107].

Of these reports and studies targeting patients with autoimmune-mediated PNS diseases, 15 reported the results of autologous peripheral blood HSCT, three reported the results of allogeneic HSCT, and two reported the results of both autologous peripheral blood and allogeneic HSCT [104,105,106,107,108,109,110,111,112,113,114,115,116,117,118,119,120,121,122,123,124]. Autologous peripheral blood hematopoietic stem cells were mobilized using cyclophosphamide + G-CSF (*n* = 13), G-CSF only (*n* = 2), vinorelbine + G-CSF (*n* = 1), and rituximab + G-CSF (*n* = 1) [104,105,106,107,108,109,110,111,112,113,114,115,116,117,118,119,120,121]. Three reports did not describe how stem cells were mobilized into the peripheral blood [122,123,124]. Peripheral blood hematopoietic stem cells were intravenously infused after application of different conditioning regimens. Most commonly, cyclophosphamide-based regimens were used (cyclophosphamide + ATG ± rituximab ± busulfane ± fludarabine ± total body irradiation), followed by a BEAM regimen (± ATG), melphalan + lenalidomide, and busulfan + fludarabine + alemtuzumab [104,105,106,107,108,109,110,111,112,113,114,115,116,117,118,119,120,121,122,123,124]. Treatment-associated adverse events were frequently reported including infections, toxicity, and transient worsening of neurological symptoms [104,105,106,107,108,109,110,111,112,113,114,115,116,117,118,119,120,121,122,123,124]. Treatment-related deaths did not occur [104,105,106,107,108,109,110,111,112,113,114,115,116,117,118,119,120,121,122,123,124]. 

Over all these investigations, only a minority (43/108, 40%; outcome not described in 60 patients) reported beneficial effects of HSCT [104,105,106,107,108,109,110,111,112,113,114,115,116,117,118,119,120,121,122,123,124]. These beneficial effects included improvement of existing neurological deficits and ambulation, stabilization of relapsing disease activity, slowing down of progressive disease, reduction in dosage or frequency of immunomodulatory treatment, and even cessation of symptomatic and immunosuppressive therapies [104,105,106,107,108,109,110,111,112,113,114,115,116,117,118,119,120,121,122,123,124]. However, two patients were not beneficially affected by autologous, peripheral blood-originating stem-cell transplantation [104,112]. In one of them, a CIDP-relapse 5 years after stem-cell transplantation could not have been prevented leading to an equal neurological state compared with the time period before transplantation [104]. Similar results were reported in an MMN patient after autologous, peripheral blood-originating stem-cell transplantation, whose neurological state did not improve and who was still dependent of monthly IVIG infusions [112].

However, a recently published phase I/II study post hoc analysis investigated the cost effectiveness of IVIG treatment and HSCT in patients with CIDP [125]. The authors emphasized that autologous HSCT is more cost-effective than long-term IVIG treatment due to the long-term treatment-free remission and better outcome measurements observed in their study [125]. Nevertheless, the authors indicated the need to consider patient selection, the stem-cell regimen, and regional variations in the cost and effectiveness analysis in future studies [125].

One patient with myasthenia gravis and concomitant ALS was treated with intrathecally and intramuscularly applied, bone marrow-derived autologous mesenchymal stem cells [120]. A conditioning regimen was not used, and no treatment-related adverse events other than a urinary tract infection occurred [120]. Seven months after stem-cell application, all neurological functions and cognition had improved, suggesting an underlying pure neuroimmunological disease [120].

In summary, 108 patients with autoimmune-mediated disorders of the PNS were treated with stem-cell therapies [104,105,106,107,108,109,110,111,112,113,114,115,116,117,118,119,120,121,122,123,124]. One phase I/II trial reported autologous HSCT in CIDP patients [107]. Due to the lack of randomized controlled studies including larger amounts of patients, stem-cell therapies in autoimmune-mediated PNS disorders constitute a therapeutic option for individual patients after careful discussions of risks and benefits with the patient [72]. Further investigations are needed to evaluate stem-cell therapies in this patient group.

### 3.5. Immune-Mediated Neurological Complications after Stem-Cell Therapies

Stem-cell therapies generally harbor the risk of adverse events due to their invasiveness. This is partly attributable to the treatment procedure, which often involves conditioning with myelotoxic and highly aggressive agents.

In general, stem-cell therapies might be a trigger for secondary immune-mediated diseases possibly involving every organ system. For instance, new onset of hematological and rheumatological immune-mediated diseases after autologous HSCT was reported. Hemophilia-A, factor VIII inhibitor, autoimmune hemolytic anemia (AIHA), and immune thrombocytopenia (ITP) occurred after autologous HSCT in patients initially suffering from MS, SLE, and systemic sclerosis [126,127,128,129]. Since four of these nine patients were conditioned with alemtuzumab, the new onset of secondary immunity due to alemtuzumab application rather than autologous HSCT should be considered [127,128,129,130,131]. In another patient with multiple myeloma, new onset of systemic sclerosis was diagnosed, while, in a patient with cerebral vasculitis (in X-linked lymphoproliferative disease as described above), new onset of anti-GBM-disease was diagnosed [103,126]. 

These secondary immune-mediated diseases after stem-cell transplantation regularly require immunomodulatory treatment. Nevertheless, aggressive disease courses are possible, and, despite different immunomodulatory treatment options, fatal outcome might occur [127,128]. Since many reports regarding the phenomenon of secondary immunity are available in the literature, we focused on reports involving the PNS and CNS.

The literature search revealed 45 articles reporting immunological adverse events in association with hematopoietic stem-cell therapy involving the PNS (see summary in Table 3; for more details, see Supplementary Table S3).

Several case reports described patients with brachial plexopathy and plexitis (*n* = 5), neuralgic amyotrophy (*n* = 2), and multiple lumbosacral radiculopathies (*n* = 1) [132,133,134,135]. In seven of these eight patients, autologous hematopoietic stem cells were transplanted for the treatment of MS (*n* = 1), multiple myeloma (*n* = 2), AL amyloidosis (*n* = 2), or lymphoma (*n* = 2) [132,133,134,135]. One patient with myelodysplastic syndrome received allogeneic HSCT [132]. Symptomatic and immunosuppressive treatment (methylprednisolone) led to recovery with residuals or complete recovery in all patients [132,133,134,135].

In several case reports and a retrospective case–control study, 75 patients with immune-mediated neuropathies after stem-cell transplantation were described [23,136,137,138,139,140,141,142,143,144,145,146,147,148,149,150,151,152,153,154,155,156,157,158,159]. The diagnostic criteria of CIDP were met in 19 of the patients, whereas 12 patients were diagnosed with GBS [23,136,137,138,139,140,141,142,143,144,145,146,147,148,149,150,151,152,153,154,155,156,157,158,159]. The other patients did not meet the diagnostic criteria for either CIDP or GBS and were, therefore, classified as immune-mediated polyneuropathies [23,136,137,138,139,140,141,142,143,144,145,146,147,148,149,150,151,152,153,154,155,156,157,158,159]. 

In some of these cases, immune-mediated polyneuropathy occurred simultaneously with acute or chronic graft-versus-host disease (GvHD) affecting other organ systems; thus, a clear differentiation between both disease entities was not possible [23,136,137,138,139,140,141,142,143,144,145,146,147,148,149,150,151,152,153,154,155,156,157,158,159]. The authors described these cases as GvHD with peripheral nervous system involvement or autoimmune-mediated polyneuropathy in GvHD [23,136,137,138,139,140,141,142,143,144,145,146,147,148,149,150,151,152,153,154,155,156,157,158,159]. 

In 20 articles including 70 patients, allogeneic hematopoietic stem cells were transplanted to treat mostly malignant hematological diseases (multiple myeloma, different leukemias, and myelodysplastic syndrome) [23,136,137,138,139,140,141,142,143,144,145,146,147,148,149,150,151,152,153,154]. Furthermore, three out of 70 patients received allogeneic HSCT for the treatment of aplastic anemia, while one out of 70 patients received it for osteopetrosis [136,139,142]. In contrast, three patients (out of three case reports) were treated with autologous hematopoietic stem cells (lymphoma *n* = 1 and multiple myeloma *n* = 2) [153,154,155]. Lastly, two case reports described the onset of immune-mediated peripheral polyneuropathy in two patients, who were treated with allogeneic umbilical cord blood stem cells for leukemia [156,157].

Treatment with different immunosuppressive therapies (corticosteroids, azathioprine, cyclosporine, MMF, IVIG, plasmapheresis, sirolimus, tacrolimus, rituximab, vincristine, and cyclophosphamide) resulted in complete or partial recovery or control of disease in almost all patients [32,136,137,138,139,140,141,142,143,144,145,146,147,148,149,150,151,152,153,154,155,156,157,158,159]. In two of the reported 75 patients with immune-mediated polyneuropathies following allogeneic HSCT for leukemia, the outcome was fatal [143,148].

The occurrence of myasthenia gravis after allogeneic HSCT was published in 16 case reports (with a total of 18 patients) [160,161,162,163,164,165,166,167,168,169,170,171,172,173,174,175]. Interestingly, there were two reports of patients in which onset of myasthenia gravis and concomitant polymyositis was described [165,166]. Stem cells were transplanted in these patients due to aplastic anemia (*n* = 8), leukemia (*n* = 6), lymphoma (*n* = 3), and combined immunodeficiency (*n* = 1) [160,161,162,163,164,165,166,167,168,169,170,171,172,173,174,175]. Treatment was symptomatic (pyridostigmine) and immunosuppressive (corticosteroids, azathioprine, plasmapheresis, cyclosporine, and thalidomide) [160,161,162,163,164,165,166,167,168,169,170,171,172,173,174,175]. The outcome was described in detail in only seven case reports (controlled under treatment *n* = 4, complete recovery *n* = 2, and death *n* = 1) [160,161,162,163,164,165,166,167,168,169,170,171,172,173,174,175].

Twenty-one case reports (including a total of 31 patients) were identified, which described the onset of immune-mediated CNS diseases after stem-cell therapy (Table 3 and Supplementary Table S3).

Reported were patients with acute demyelinating encephalomyelitis (ADEM) and ADEM-like syndromes (*n* = 7), immune-mediated encephalitis (*n* = 5, one each with *N*-methyl-d-aspartate-receptor (NMDAR), glutamate-decarboxylase (GAD), leucine-rich, glioma inactivated 1 (LGI1) and GAD, and contactin-associated-protein 2 (CASPR2) antibodies), immune-mediated myelopathy (*n* = 15, one with MOGAD, and one with AQP4 antibody-negative NMOSD with fulfillment of the diagnostic criteria, 13 with seronegative myelopathies; three had additional optic neuritis), MS (*n* = 3), and bilateral optic neuritis (*n* = 1) [23,136,176,177,178,179,180,181,182,183,184,185,186,187,188,189,190].

Stem-cell therapies were offered as a treatment option due to malignant diseases (leukemia, lymphoma, myelodysplastic syndrome, and pineoblastoma) in most cases and rarely due to autoimmune-mediated diseases (one each for MS, myelitis, and CIDP) [23,136,176,177,178,179,180,181,182,183,184,185,186,187,188,189,190]. Hematopoietic stem cells of different origin (allogeneic *n* = 26, autologous *n* = 1, and not described *n* = 1) were infused in all but two patients [23,136,176,177,178,179,180,181,182,183,184,185,186,187,188,189,190]. In these two patients, allogeneic umbilical cord blood-derived mesenchymal stem cells and autologous bone marrow-derived mesenchymal stem cells (myelitis) were applied for the treatment of autoimmune-mediated diseases [189,190].

These immune-mediated adverse events affecting the CNS were addressed with immunomodulatory treatment including corticosteroids, plasmapheresis, IVIG, cyclophosphamide, cyclosporine A, tacrolimus, ATG, rituximab, and interferon beta [23,136,176,177,178,179,180,181,182,183,184,185,186,187,188,189,190]. Complete recovery was rarely achieved in these patients (*n* = 6), while most patients were treatment-dependent, although disease activity was controlled under treatment [23,136,176,177,178,179,180,181,182,183,184,185,186,187,188,189,190]. The outcome was fatal in four out of 31 reported patients [23,136,176,177,178,179,180,181,182,183,184,185,186,187,188,189,190].

In all these patients with immune-mediated PNS and CNS pathologies after stem-cell therapies, a possible causative role of the chosen stem-cell mobilization procedure, conditioning regimen, or GvHD prophylaxis must be considered. However, only a small percentage of the respective articles reported these data. According to the cases reported in the literature so far, the occurrence of secondary immunity against neuronal tissue is particularly associated with allogeneic hematopoietic stem-cell transplantation.

## 4. Conclusions

Several studies indicated the beneficial effects of different variants of stem-cell transplantation in various neurological autoimmune disorders. In phase I/II and II studies of MS, NMOSD, and CIDP, the efficacy and the safety of transplantation of hematopoietic and mesenchymal stem cells were reported. Nevertheless, severe treatment procedure-related adverse events were frequently reported, including death of the treated patients.

Compared with the high number of stem-cell transplanted patients, secondary immunity affecting the CNS and PNS after stem-cell transplantation is rare. However, due to the complexity of these patients, there may be an underreporting bias. HSCT might play a crucial role in the future treatment of MS. Despite the large number of available DMTs for MS, several studies including phase III trials are currently being conducted to directly compare autologous HSCT with existing immunotherapies. 

Due to the lack of clinical trials combined with the now available and emerging DMTs, the role of stem-cell therapy in other neurological autoimmune disorders is currently limited to the treatment of refractory patients with poor prognosis.

## Figures and Tables

**Table 1:** Stem-cell therapies in multiple sclerosis.

Applied Cells	First Author	Reference	Patients (*n*)	MS Type (*n*)			Age	Females (*n*)	EDSS (before Treatment)	Follow-Up Duration (Months)	Outcome (Beneficial Effects) ****, %
				PPMS	RRMS	SPMS					
**HSC (auto)**	Burt	17	52		52		35.6 **	34	3.4 **	33.6 **	93
	Gale	46	426		ND		47 *	281	ND	ND	ND
	Moore	45	35		20	15	37 *	24	6 *	36 *	37
	Ruiz-Arguelles	44	617	130	259	228	46 *	401	5.5 *	3–42	18
	Ruiz-Arguelles	43	286	62	110	114	47 *	194	5 *	3 **	16
	Atkins	42	24		12	12	34 *	14	4.9 **	80.4 **	71
	Mancardi	41	9		2	7	36 *	5	6.5 *	48 **	100
	Nash	40	25		25		37.3 **	17	4.4 **	46.5 **	69
	Berard	38	23		12	11	32.65 **	14	4.87 **	36 **	ND
	Walker	39	7		7		37.4 **	ND	4.64 **	24 **	ND
	Shevchenko	37	95	15	45	35	34.5 **	59	3.5 *	46 **	100
	Hamerschlak	36	41	4	4	33	42 **	24	6.5 *	36 **	59
	Burt	35	21		21		33 **	11	3.1 **	37 *	100
	Capello	34	21		4	17	36 *	ND	6.5 *	ND	95
	Saccardi	33	19		4	15	36 *	12	6.5 *	36 *	95
	Saiz	32	14		5	9	30 *	12	6 *	36 *	86
	Burt	31	21	6	1	14	38.9 **	10	6.4 **	26 **	62
	Espigado	29	22	Progressive MS (ND)			ND	ND	ND	10 *	77
	Nash	30	26	8	1	17	41 *	12	7 *	28 *	77
	Mancardi	28	10			10	37.1 **	5	6.5 *	15 *	60
	Saiz	27	5		2	3	33.6 **	4	6.5 *	18 *	60
	Fassas	26	24	8		16	40 *	12	6 *	40 *	75
	Kozak	25	11			11	35.7 **	9	6.7 **	8.5 *	91
	Fassas	24	15	8		7	37 *	7	6 *	6 *	93
**MSC (auto)**	Uccelli	62	144	17	94	33	39 **	87	4 *	5.6 ***	87
	Petrou	61	48	7		41	47.63 **	21	5.6 **	12 ***	35
	Sahraian	60	4		1	3	28 **	1	4.25 **	24 ***	75
	Cohen	57	25		11	14	46.4 **	17	6 *	6 ***	72
	Fernandez	65	19			19	46 **	13	7.5 *	12 ***	100
	Harris	59	20	4		16	49 **	14	6.8 **	6 ***	40
	Dahbour	58	10		2	8	34.9 **	4	5.1 **	12 ***	60
	Harris	56	6	2		4	42.7 **	4	7.3 **	88.8 **	100
	Stepien	64	20		13	7	38 **	8	5 *	18 ***	100
	Llufriu	55	9		9		36.8 **	7	3.5 *	13 *	89
	Bonab	54	25	2		23	37.7 **	19	6.1 **	12 ***	16
	Connick	53	10			10	48.8 **	3	6.1 **	18 ***	ND
	Hammadi	63	50	Progressive MS (ND)			44 **	25	7	12 ***	48
	Karussis	52	15	Progressive MS (ND)			35.3 **	8	6.7 **	6–25	100
	Yamout	51	10		1	9	38.5 **	6	4-7.5	12 ***	70
	Bonab	50	10	2		8	33 **	77	3.5–6	19 **	60
**MSC (allo)**	Roirdan	68	20	4	15	1	41.15 **	12	5.23 **	12 ***	100
	Li	66	13	RRMS/SPMS (ND)			41.7 **	9	6.98 **	12 ***	ND
	Lublin	67	16		10	6	48 *	11	4.8 **	12 ***	94
**Peptide-loaded tolerogenic** **dendritic cells (auto)**	Zubizarreta	9	8	3	1	4	49.25 **	4	5.9 **	3 ***	100

MS = multiple sclerosis; PPMS = primary progressive MS; RRMS = relapsing–remitting MS; SPMS = secondary progressive MS; EDSS = expanded disability status scale; HSC = hematopoietic stem cell; MSC = mesenchymal stem cell; auto = autologous; allo = allogeneic; * = median; ** = mean; *** = predefined follow-up duration; **** = improvement in or stability of EDSS scores during follow-up; ND = no data.

**Table 2:** Stem-cell therapies in NMOSD.

Applied Cells	First Author	Reference	Patients (*n*)	AQ4 IgG-Positive	Age	Females (*n*)	EDSS (before Treatment)	Follow-Up Duration (Months)	Outcome (Beneficial Effects) *****, %
**HSC (auto)**	Burton	79	3	2	34 **	2	4 *	108	67
	Khan	85	1	1	2	1	ND	6	0
	Carlisle	80	1	1	40	1	ND	24	100
	Burt	78	13	12	42 **	11	4.3 **	57	80 ****
	Aouad	82	1	1	47	1	6.5	12	100
	Greco	77	16	10	37 *	13	6.5 *	47	88
	Hoay	81	3	ND	31.7 **	1	3.5 *	88	100
	Matiello	83	1	1	64	1	ND	24	0
	Peng	84	1	ND	23	1	5	6	100
**HSC (allo)**	Hau	88	1	1	15	0	8.5	48	100
	Ceglie	86	1	1	9	1	6.5	24	100
	Greco	87	2	1	29 **	1	7.5 *	42	100
**MSC (auto)**	Fu	91	15	13	47 **	14	4.9 **	24	100
**MSC (allo)**	Lu	89	5	5	25.4 **	5	5 *	70	60
	Lu	90	5	5	35 **	5	5.1 **	24	80
**Peptide-loaded tolerogenic** **dendritic cells**	Zubizarreta	9	4	4	40 **	3	4.8 **	3	100

NMOSD = neuromyelitis optica spectrum disorders; AQP4 IgG = aquaporin-4 immunoglobulin G antibody; EDSS = expanded disability status scale; HSC = hematopoietic stem cell; MSC = mesenchymal stem cell; auto = autologous; allo = allogeneic; * = median; ** = mean; **** considering 12 patients (excluding one patient with NMOSD and concomitant systemic lupus erythematosus); ***** = improvement or stability of EDSS scores during follow-up; ND = no data.

**Table 3:** Immune-mediated neurological diseases after stem-cell therapies.

Immune-Mediated Neurological Diseases	Publications (*n*)	Patients (*n*)	Females (*n*)	Age (Min–Max, Years)
Immune-mediated neuropathies	29	83	28	0.6–69
Myasthenia gravis (± polymyositis)	16	18	7	3–54
Encephalitis/myelitis	21	31	12	6–64

Immune-mediated neuropathies = chronic inflammatory demyelinating polyneuropathy, acute inflammatory demyelinating polyneuropathy, Guillan–Barré syndrome, inflammatory neuropathies, and plexopathies; encephalitis/myelitis = multiple sclerosis, acute demyelinating encephalomyelitis, neuromyelitis optica spectrum disorder, myelin oligodendrocyte glycoprotein-associated disease, leucine-rich glioma-inactivated 1 antibody encephalitis, *N*-methyl-d-aspartate-receptor antibody encephalitis, glutamate-decarboxylase antibody encephalitis, contactin-associated protein 2 antibody encephalitis, optic neuritis, Bickerstaff encephalitis, and myelitis.

## Data Availability

The datasets used and/or analyzed during the current study are available from the corresponding author on reasonable request.

## Supplementary Materials

The following supporting information can be downloaded at https://www.mdpi.com/article/10.3390/cells11142165/s1. **Table S1.** Stem cell therapies in multiple sclerosis. MS = multiple sclerosis; PPMS = primary progressive MS; RRMS = relapsing–remitting MS; SPMS = secondary progressive MS; EDSS = expanded disability status scale; HSC = hematopoietic stem cell; MSC = mesenchymal stem cell; BEAM = carmustine, etoposide, cytarabine, and melphalan; ATG = anti-thymocyte globulin; i.v. = intravenous; i.t. = intrathecal; * = median; ** = mean; *** = predefined follow-up duration; ND = no data; **Table S2.** Stem-cell therapies in NMOSD. c = case report/case series; NMOSD = neuromyelitis optica spectrum disorder; AQP4 IgG = aquaporin-4 immunoglobulin G antibody; EDSS = expanded disability status scale; HSC = hematopoietic stem cell; MSC = mesenchymal stem cell; BEAM = carmustine, etoposide, cytarabine, and melphalan; ATG = anti-thymocyte globulin; i.v. = intravenous; i.t. = intrathecal; * = median; ** = mean; **** considering 12 patients (excluding one patient with NMOSD and concomitant systemic lupus erythematosus); ***** = improvement or stability of EDSS scores during follow-up; ND = no data; **Table S3.** Autoimmune-mediated complications of stem-cell therapies affecting the central and/or peripheral nervous system. PNS = peripheral nervous system; CNS = central nervous system; MS = multiple sclerosis, NMOSD = neuromyelitis spectrum disorder; AIDP = acute inflammatory demyelinating polyneuropathy; CIDP = chronic inflammatory demyelinating polyneuropathy; GBS = Guillain–Barré-syndrome; (c) GvHD = (chronic) graft-versus-host disease; ADEM = acute demyelinating encephalomyelitis; MG = myasthenia gravis; GAD = glutamine acid decarboxylase; NMDAR = *N*-methyl-d-aspartate receptor; LGI1 = leucine-rich glioma-inactivated 1; MOGAD = myelin oligodendrocyte glycoprotein-associated disease; HSC = hematopoietic stem cell; UCB = umbilical cord blood; MSC = mesenchymal stem cell; ** = mean; ND = no data.
